# Dynamic Changes in Gene Mutational Landscape With Preservation of Core Mutations in Mantle Cell Lymphoma Cells

**DOI:** 10.3389/fonc.2019.00568

**Published:** 2019-07-03

**Authors:** Qian Zhang, Hong Y. Wang, Xiaobin Liu, Michael H. Roth, Alex A. Shestov, Seung-Cheol Lee, Kanika Jain, Craig Soderquist, Qun-Bin Xiong, Marco Ruella, Honore Strauser, Jerry D. Glickson, Stephen J. Schuster, Andrzej Ptasznik, Mariusz A. Wasik

**Affiliations:** ^1^Department of Pathology and Laboratory Medicine, Abramson Cancer Center, Perelman School of Medicine of the University of Pennsylvania, Philadelphia, PA, United States; ^2^Department of Radiology, Abramson Cancer Center, Perelman School of Medicine of the University of Pennsylvania, Philadelphia, PA, United States; ^3^Department of Lymphoma Program, Abramson Cancer Center, Perelman School of Medicine of the University of Pennsylvania, Philadelphia, PA, United States; ^4^Department of Pathology, Fox Chase Cancer Center, Philadelphia, PA, United States

**Keywords:** mantle cell lymphoma, Bruton's tyrosine kinase, histone 3/lysine 4 (H3K4) demethylase, ibrutinib, KDM5C

## Abstract

While studies have identified a number of mutations in mantle cell lymphoma (MCL), the list may still be incomplete and contribution to the pathogenesis remains unclear. We analyzed the mutational landscape of four mantle cell lymphoma biopsies obtained during an 8-year period from the same patient with his normal cells serving as control; we also established a cell line from the final stage of the disease. Numerous mutations with high allelic burden have been identified in all four biopsies. While a large subset of mutations was seen only in individual biopsies, the core of 21 mutations persisted throughout the disease. This mutational core is also maintained in the cell line that also displays DNA-methylation and cytokine secretion profiles of the primary mantle cell lymphoma cells. This cell line is uniquely sensitive to clinically relevant inhibitors of Bruton's Tyrosine Kinase. The response to Bruton Tyrosine Kinase's inhibition is enhanced by inhibitors of CDK4/6 and mTOR. Among the mutations seen in the primary and cultured MCL cells, mutations of three genes are involved in the control of H3K4 methylation: demethylase KDM5C, present already in the early disease, and methyltransferase KMT2D and cofactor BCOR, both of which are seen late in the disease and are novel and predicted to be pathogenic. The presence of these mutations was associated with hypermethylation of H3K4. Restoration of KDM5C expression affected expression of numerous genes involved in cell proliferation, adherence/movement, and invasiveness.

## Introduction

Mantle cell lymphoma (MCL), a B-cell lymphoma with t(11:14) translocation involving the CyclinD1 (CCND1) gene (1), has typically poor prognosis (2). Introduction of novel therapies for MCL is restricted by a still poor understanding of the lymphoma pathogenesis and unclear adequacy of experimental models. DNA sequencing studies (3–7) in MCL identified diverse gene mutations; only a few affecting ATM, CCND1, and KMT2D (MLL-2) displayed a relatively high frequency. However, the pathogenic role of these and other less frequent mutations remains to be elucidated. Although patient-derived cell lines are widely used to study MCL and cancer in general, their relationship to primary malignant cells has not typically been adequately determined with the exception of the MCL-derived CCMCL1 line in which a relatively comprehensive molecular analysis was performed (8).

Here we report that whole-exome sequencing (WES) of four different MCL specimens obtained from the same patient at different times identified a striking gene mutational pattern. While many of the tumor-specific, high-allelic frequency mutations were seen at only a single time point, 21 other mutations, many novel, persisted throughout the entire disease. These core mutations were preserved in a cell line we developed at late stage of the lymphoma. The cell line matched also the cytokine-secretion and DNA methylation profiles of the primary cells and displayed high sensitivity to clinically relevant inhibitors of Bruton's tyrosine kinase (BTK) (9, 10), CDK4, and mTOR. Among the identified mutations, we have focused particularly on the novel and persistent mutation of H3K4 demethylase KDM5C and its consequences. Implications of our findings are discussed.

## Materials and Methods

### Primary MCL Cells and Establishment of MCL-RL Cell Line

The patient was diagnosed in 2003 with high stage MCL involving multiple lymph nodes and the colon received numerous and diverse therapies until 2013, when he developed central nervous system involvement and pleural effusions containing numerous lymphoma cells. The history of the patient's biopsies and treatment modalities is schematically depicted in the Supplemental Figure 1.

The therapies comprised of Rituximab (R; administered in 2005), R-CHOP (2007), radiation (2008), R-bendamustine (2009 and 2012), bortezomib and dexamethasone (2012), radiation (2013), Ibrutinib (2013), R-hCEV (2013), Revlimid and Cytoxan (2013). We analyzed a mutational landscape of MCL tissue biopsies from the same patient obtained in 2005, 2007, 2009, and pleural effusion exudate from 2013 with the patient's normal peritoneal mesothelial cells serving as a control; we also managed to establish a cell line from the 2013 sample representing the most advanced disease stage of the disease. All samples shared the same CD20+/CD5+/CCND1+ cell phenotype; the three earlier ones were analyzed using formalin-fixed, paraffin-embedded tissues, and the 2013 sample was a pleural effusion exudate comprised in >95% of MCL cells. The studies were performed according to the institutional biosafety standards under the IRB-approved protocol (826234).

### Standard MCL Cell Lines

JeKo-1 was from the American Type Culture Collection (ATCC), while Mino and SP49 cells were obtained from Dr. Lai, University of Alberta. Cell identity was confirmed by flow cytometric and IgH/CCND1 fusion FISH evaluation. Cells were grown in RPMI with 10% fetal bovine serum, 1% L-glutamine, and 1% penicillin/streptomycin.

### Whole Exome DNA Sequence Analysis

DNA isolated using a DNeasy Tissue kit (Qiagen) underwent whole exome sequence analysis at the Children Hospital of Philadelphia core facility served by the Beijing Genomic Institute (BGI). Whole exome library was prepared using Agilent SureSelect Whole Exome v. 5 sequencing kit (Agilent, Santa Clara, CA) and, for formalin-fixed paraffin-embedded samples, also Kapa Hyperprep kit (Roche, Basel, Switzerland). The library construction included DNA fragmentation, end repair and A-tailing, ligation, amplification, and hybridization of capture probes, with intervening washes and purification steps as required. The resulting library is typically 300–400 bp; library size, quality and concentration were assessed using a LabChip GX instrument (Perkin Elmer, Waltham, MA) and/or by qPCR. Indexed libraries were pooled and 100 ×100 bp paired-end 100 bp sequencing was performed on a Hiseq 4000 platform (Illumina, San Diego, CA).

### Exome Sequencing Alignment and Variants Calling With Annotation

After removal of low-quality base and trimmed adapter sequences by Cutadapt, the unaligned reads in FASTQ format were first filtered by Bowtie 2 for contaminants and evaluated by Pre-alignment QA/QC, then aligned to the hg19 reference genome using Bowtie 2, TopHat 2.0, and STAR. Using Partek Flow pipeline variants in aligned reads were called by Sam tools Mpileup 1.4.1 on all samples, annotated with SnpEff, VEP and Ensembl Transcripts 75 and confirmed using IGV and GoldenHelix sequence browsers. Variants were filtered by reads number (cutoff at 10) and high allelic frequency (cutoff at 20% for fixed non-malignant cell-rich nodal tissue and 40% for fresh/frozen essentially pure malignant cell populations). Benign single nucleotide polymorphisms (SNPs) were excluded using ExAc, 1000 Genomes, and gnomAD databases. Polymorphisms identified in the patient's normal cells were also excluded to identify mutations acquired by malignant cells. Missense mutations of high interest were confirmed by pyrosequencing; a non-sense mutation by Western blot to document the loss of protein expression.

### Whole Genome DNA Methylation Analysis

DNA isolated using a DNeasy Tissue kit (Qiagen) was analyzed at Zymo Research (Irvine, CA). Libraries were prepared from digested genomic DNA and extracted with a DNA Clean & Concentrator™-5 kit. Fragments were ligated to pre-annealed adapters containing 5′-methyl-Cytosine, recovered from NuSieve /agarose gel, and treated with bisulfite. Preparative-scale PCR was performed and the resulting products were purified and sequenced on an Illumina HiSeq. Sequence reads from bisulfite-treated EpiQuest libraries were identified using standard Illumina base-calling software and then analyzed using a Zymo Research proprietary analysis pipeline written in Python; Bismark was used to perform the alignment. Filled-in nucleotides were trimmed off during methylation calling. Targeted DNA sequence analysis. Genomic DNA and cDNA were PCR-amplified using biotinylated primers specific for exon 1 of CCND1a and b isoforms and proximal promoter/exon1 region of B2M gene. Purified PCR products were rendered single-stranded on a Pyrosequencing AB workstation and annealed to the gene-specific sequencing primers. Quantitative DNA mutational analysis was performed on a PSQ 96MA system with the PyroGold SQA reagent kit (Pyrosequencing AB) and results were analyzed using the Q-CpG software.

### Scoring of Gene Mutation Oncogenicity

Two state-of-the-art algorithms were used to determine the oncogenic nature of mutations: the FATHMM-MKL algorithm with results reported in a COSMIC database was used to evaluate missense mutations and Mutation Taster for non-sense mutations. Prediction scores are given in the range from 0 to 1 with scores > 0.5 predicted to be pathogenic. These are currently the two most accurate algorithms predicting the functional effects of protein mutations and the values close to the extremes (0 or 1) are the highest confidence predictions that yield the highest accuracy.

### RNA Sequence (RNASeq) Analysis

RNASeq was performed using Illumina's truSeq riboZero stranded library prep kit. The libraries were sequenced on Illumina HiSeqs 2500 using V4. Reads were trimmed to remove low-quality sequences and then aligned to the genome (Human hg19) and quantified on RefSeq transcripts using the RNA-Seq Unified Mapper (RUM). Length normalized counts (FPKM counts) were extracted from the RUM output. Pairwise comparisons between groups were carried using a custom script that implemented Bioconductor software's package edgeR to compute a *p*-value and fold-change for each transcript.

### Flow Cytometry

MCL cells incubated with the PE-conjugated anti-human MHC class I and MHC class II antibodies or control IgG antibody (R&D Systems) were analyzed using flow cytometry (FACSCalibur; BD Biosciences) and CellQuest Pro software.

### Soluble Cytokine/Receptor Detection

Cryopreserved (−80°C) samples were analyzed using Human cytokine 30-plex panel (Life Technologies, Carlsbad, CA) and FlexMAP 3D instrument (Luminex, Austin, TX). Data analysis was done using xPONENT software (Luminex).

### Enzyme Immunoassay (EIA)

IL-6 and IP-10 supernatant concentrations were detected using EIA kits (Sigma). Supernatants from 24 h cultures were deposited into plastic well strips pre-coated with an anti-IL-6 or anti- IP10 antibody. The second set of cytokine-specific antibodies conjugated to peroxidase was added and colorimetric substrate conversion evaluated on EIA plate reader.

### MTT Enzymatic Conversion Assay

Cells were plated in 96-well plates (3–5 ×10^3^/well), incubated with drugs or their vehicles, labeled with MTT (Promega) and evaluated using a Titertek Multiskan reader. DNA fragmentation (TUNEL) assay. MCL cells treated with drugs or their vehicles and fixed for TUNEL labeling (Roche) were analyzed by flow cytometry (BD Biosciences); the data were analyzed with flowjo software.

## Results

### Gene Mutational Landscape in Consecutive Biopsies of MCL

**Figure 1:**
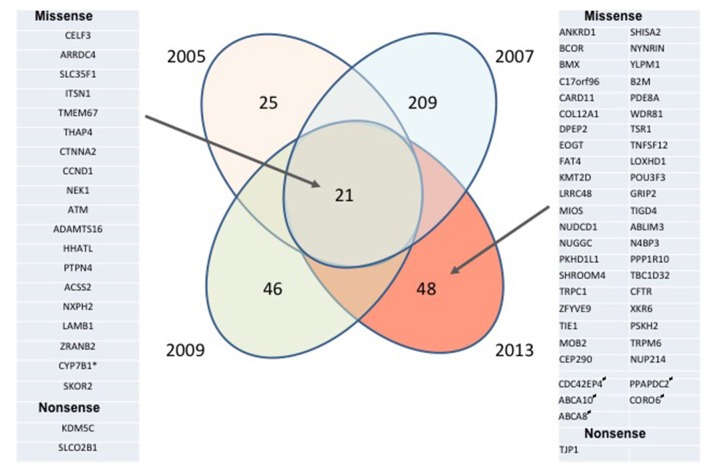
Longitudinal analysis of mutational landscape in MCL samples. MCL samples collected from the same patient in 2005, 2007, 2009, and 2013 and his normal peritoneal mesothelial cells were analyzed by WES; the depicted mutation numbers reflect tumor-specific mutations with high allelic frequency (≥ 20% for formalin-fixed 2005, 2007, and 2009 tissue biopsies and ≥ 40% for the unfixed 2013 pleural exudate comprised in >95% of MCL cells). Mutations preserved throughout the disease duration are listed on the left, mutations seen only in 2013 are listed on the right. *Absent in 2005 sample; ^#^Monoallelic germline mutation, “biallelic” (most likely due to the loss of heterozygosity) in malignant cells.

To gain insight into gene mutations potentially involved in MCL development and progression, we performed WES of four lymphoma biopsies obtained from the same patient 2–4 years apart using the patient's normal cells as a control. A large number of malignant cell-specific mutations have been identified in each sample, even when only mutations with high allelic burden were considered (Figure 1 and Supplemental Table 1) to identify potential drivers of disease development and progression. Strikingly, the mutations fell into two broad categories: biopsy/disease stage-associated and common for essentially all biopsies. Mutations were seen at a high allelic frequency in only one of the biopsies, which likely reflected emergence and loss of dominant sub-clones of the lymphoma. The most dramatic example of this fluctuation is the 2007 biopsy containing a very high number (>200) of such sample/disease stage-associated mutations; none of them were detected as frequently at the later stages of the disease, indicating the dominant sub-clone eradication by a multi-agent immuno-chemotherapy (R-CHOP) applied at that time. The second group was comprised of 21 common mutations that were present in essentially all biopsies, possibly reflecting the set of core mutations required to sustain the malignant nature of this MCL.

### MCL-Derived Cell Line Preserves and Maintains Mutational, Soluble Protein Expression, and DNA Methylation Profile of Primary Cells

Of note, all these core mutations as well as an additional 48 mutations seen only in the advanced disease were present and maintained in a long-term MCL-RL cell line culture (Supplemental Table 1), which we succeeded in developing from the 2013 malignant pleural exudate. The cell line recapitulated very faithfully the primary 2013 MCL cells also in the other evaluated aspects: cytokine secretion and genome-wide DNA methylation. Indeed, the exudate with its cellularity was comprised of numerous MCL cells, and the MCL-RL cell line supernatant displayed very similar 30 cytokine/cytokine receptor profiles, with the exception of eotaxin and IL-5, both of which were present at much higher concentrations in the supernatant (Supplemental Table 2). While MCL-RL shared to a large extent the cytokine/receptor expression pattern with the other MCL cell lines, some cytokines/receptors were distinctly and strongly expressed by MCL-RL including soluble (s)IL-2Ra, IL-6, and IL-8. However, even these cytokines are frequently increased in the blood of MCL patients, with some, including sIL-2Ra and IL-8, being recognized as adverse prognostic factors (11, 12) We have validated increased expression by MCL-RL of IL-6 and IP-10, by standard EIA (Supplemental Figure 2). These cytokines may serve as biomarkers of tumor burden *in vivo*, since MCL-RL cells, both cultured (10) and primary (13), grow in immunodeficient mice. The DNA methylation profiles of the CpG sites throughout the entire genome were essentially identical in the MCL primary and cultured cells, regardless of the length of time in culture of the latter (Supplemental Figure 3). Although some shifts were noted (Supplemental Table 3), they affected <1% of the CpG sites when a cutoff of 50% change in methylation was applied (to account for population-wide change in methylation of the CpG site on one allele) and <0.05% sites with a 70% change cutoff (to account for the combination of allele methylation and loss or, less likely, altered methylation status on both alleles). Importantly, these rather rare changes were randomly distributed throughout the entire genome without any clustering noted or commonality between the cell line sub-lines and length of culture, possibly reflecting methodological rather than biological variability. Accordingly, analysis of gene promoter regions, where arguably DNA methylation matters most as it is able to silence genes completely, revealed only 3–7 CpG sites with 50% methylation change and 0–2 CpG sites with 70% change among 6,665 CpG analyzed (Supplemental Table 5). Similar to whole genome methylation, these rare promoter methylation changes did not show discernable patterns, as shown for the set of 228 genes (Supplemental Table 4).

### MCL-RL Cell Line Displays Clinically Relevant Kinase Activation and Inhibition Pattern

**Figure 2:**
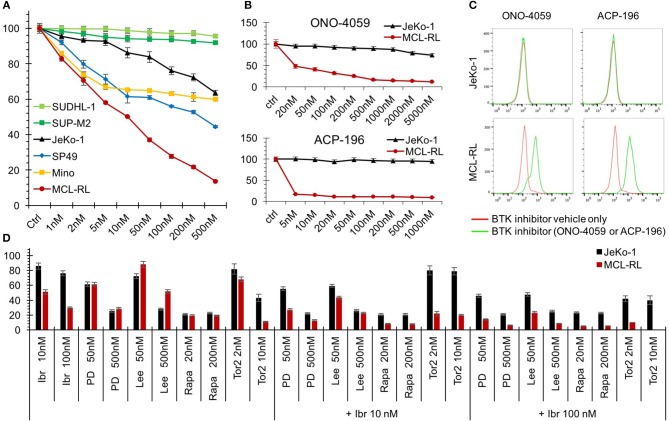
Sensitivity of MCL-RL cells to clinically relevant kinase inhibitors. **(A)** Evaluation of cell growth inhibition of MCL cells by BTK inhibitor Ibrutinib. MCL cell lines MCL-RL, Mino, SP49, and JeKo-1 were cultured for 72 h in the presence of the depicted doses of Ibrutinib and evaluated for growth inhibition using MTT conversion assay. T-cell lymphoma cell lines expressing NPM-ALK chimeric kinase SUP-M2 and SUDHL-1 served as negative controls. The results are depicted as the percentage of response with the cells cultured in medium containing drug vehicle alone (Ctrl) serving as the reference value (100% response). **(B)** Evaluation of MCL cell growth inhibition by second-generation BTK inhibitors ONO-4059 and ACP-196. MCL-RL and JeKo-1 cells were cultured for 48 h in the presence of the depicted doses of the inhibitors and analyzed for growth inhibition in the MTT conversion assay. The results are depicted as the percentage of response with the cells cultured in medium containing drug vehicle alone (Ctrl) serving as reference (100% response). **(C)** Analysis of BTK inhibition effect on apoptotic cell death. JeKo-1 and MCL-RL cells were cultured for 72 h with either ONO-4059 or ACP-196 BTK inhibitor (green lines) or drug vehicle alone (red lines) and examined for DNA fragmentation in the TUNEL assay. **(D)** Evaluation of MCL cell growth inhibition by clinically relevant kinase inhibitors. JeKo-1 (black columns) and MCL-RL (red columns) cells were cultured for 48 h at the depicted drug doses with BTK inhibitor ibrutinib (Ibr), CDK4/6 inhibitors PD0332991 (PD), or Lee011(Lee), or indirect mTORC1 inhibitor rapamycin (Rapa) or direct mTOR inhibitor Torin2 (Tor2), either alone or in combination with ibrutinib. The results are depicted as the percentage of response with the cells cultured in medium containing drug vehicle alone serving as reference (100% response).

Of note, our MCL-RL cell line displayed high sensitivity to inhibitors of BTK when compared to standard MCL cell lines (Figure 2). FDA-approved ibrutinib (1, 2, 8, 9) strongly suppresses the growth of MCL-RL, while it exerts a limited effect on SP49, Mino and Jeko-1 lines (Figure 2A). The second-generation BTK inhibitors (14), FDA-approved acalabrutinib and investigational ONO-4059, also strongly and selectively suppress the growth of MCL-RL cells but not Jeko-1 cells (Figure 2B); they also induce apoptotic cell death of the former but not the latter (Figure 2C). MCL-RL cells are also highly sensitive to inhibitors, used either as single agents or in combination with inbutinib (Figure 2D), of two other clinically relevant kinases: CDK4/6 and mTOR kinase (15–19). While CDK4/6 and mTOR inhibition alone was also effective in JeKo-1 cells, addition of ibrutinib was not beneficial, indicating that resistance to ibrutinib cannot be easily overcome by blocking additional signaling pathways.

### Characterization and Oncogenic Potential of the Identified Mutations

To potentially achieve an insight into a potential pathogenic role of the high allelic frequency mutations seen in all samples as well as the distinct ones identified in only the last 2013 sample and the derived MCL-RL cell line, we applied computational algorithms FATKM-MKL and Mutation Taster to determine oncogenic potential of the identified mutations and reviewed COSMID and other databases. Of the 69 mutated genes identified by us (Figure 1; Supplemental Table 1), 14 have been reported before as in MCL, mutations of the other ones have been seen in other hematopoietic cell malignancies, in particular, other types of B- and T-cell lymphoma. While the vast majority (64) of the mutations are absent in normal cells, five are mono-allelic (frequency >40%) in normal cells and bi-allelic in the malignant cells (frequency >85%), unless the loss of the normal allele had occurred. It remains to be determined whether these mutated genes—none of them reported as begin allelic variations so far—are recessively inherited proto-oncogenes. Thirty-one of the mutations not reported in MCL before are predicted to be pathogenic, passed on the predictive algorithms.

**Figure 3:**
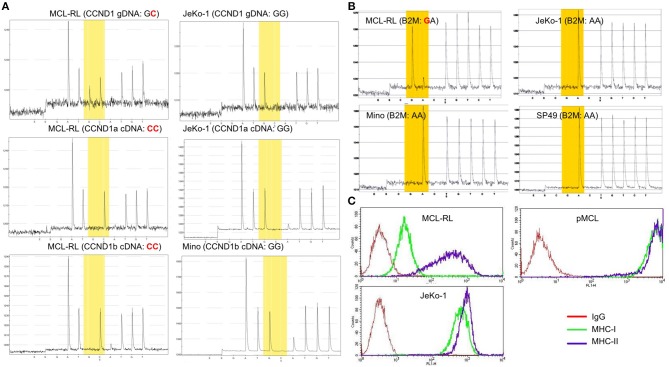
Mutational status of CCND1 and B2M in primary and cultured MCL cells. **(A)** Analysis of CCND1 gene C47S point mutation and mutant mRNA expression in MCL-RL cells. CCND1 genomic (g) and complementary (c) DNA of the CCND1a and CCND1b splice variants from first MCL-RL sub-cell line were analyzed by pyrosequencing using sequencing primers specific for the gene's first exon 1. CCND1 gDNA and splice variant a cDNA from JeKo-1 cell line and splice variant b cDNA from Mino line served as non-mutated (wild-type) gene sequence controls. **(B)** ß2-macroglobulin (B2M) M1V point mutation in MCL-RL cells. gDNA was analyzed by pyrosequencing using sequencing primers specific for the proximal promoter/exon 1 region of the B2M gene. B2M gDNA from MCL cell lines JeKo-1, Mino, and SP49 served as non-mutated (wild-type) gene sequence controls. **(C)** Analysis of B2M-dependent MHC class I expression in MCL-RL cells. MCL-RL cell line was stained with antibodies against MHC class I (MHC-I), MHC class II (MHC-II) or control IgG and examined by flow cytometry. JeKo-1 cell lines and circulating MCL cells from an MCL patient (pMCL) served as positive controls.

In regard to the 21 gene mutations seen in essentially all MCL samples including the MCL-RL cell line, a few are well-recognized and relatively frequent in MCL, representing the foremost mutations of genes encoding ATM and CCND1(3–6). However, the specific ATM missense mutation we have identified (p.I2899L) is novel for malignancy of any kind and highly oncogenic (score: 0.994), as per the FATHMM-MKL algorithm. Of note, we have also identified in all the samples mutation of NEK1, another gene involved in DNA damage repair (20), similar to ATM. This NEK1 mutation (p.D753G) is also novel and expected to be pathogenic (score: 0.871). While no mutations of the NEK1 gene have been reported so far in MCL, they have been seen in Sezary Syndrome, chronic lymphocytic leukemia, and other hematopoietic malignancies. The CCND1 mutation (C47S), one of the cluster mutations already reported in MCL, is also seemingly oncogenic (score: 0.795). We have confirmed the presence of WES-identified CCND1 mutation in MCL-RL cells using gene-specific pyrosequencing of genomic and complementary DNA (Figure 3A). Noteworthily, while the CCND1 mutation is mono-allelic, only the mutated gene is expressed, as seen in both CCND1a and CCND1b splice variant forms (14). The selective expression of the CCND1^**^ mutant was also identified using RNASeq (Supplemental Table 1). These findings indicate that the mutation occurred at approximately the same time as translocation of the CCND1 gene since fusion with immunoglobulin gene promoter is required for expression of CCND1 mRNA.

Among the 48 mutations seen only in the 2013 primary and cultured cells, there was a mutation of ß2-macroglobulin gene (p.M1V), reported previously in MCL and other types of lymphoma. We have confirmed the presence of this mutation by pyrosequencing (Figure 3B) and showed that it was associated with a greatly diminished expression of MHC class I protein (Figure 3C), indicating role of the mutation in immune evasion. Two other mutated genes in this group (WDR81 and TRPC1) are involved in regulating PI3 kinase-mediated signaling pathways. While no mutations of these genes have been reported so far in MCL, their mutations have been seen in lymphomas and acute myeloid leukemia. However, specific mutations of WDR81 (p.A223T) andTRPC1 (p.L52F) identified by us are novel for malignancy of any kind and, in addition, pathogenic with scores of 0.894 and 0.956, respectively.

### Mutation of KDM5C and Other Genes Involved in the Control of H3K4 Methylation Status

**Figure 4:**
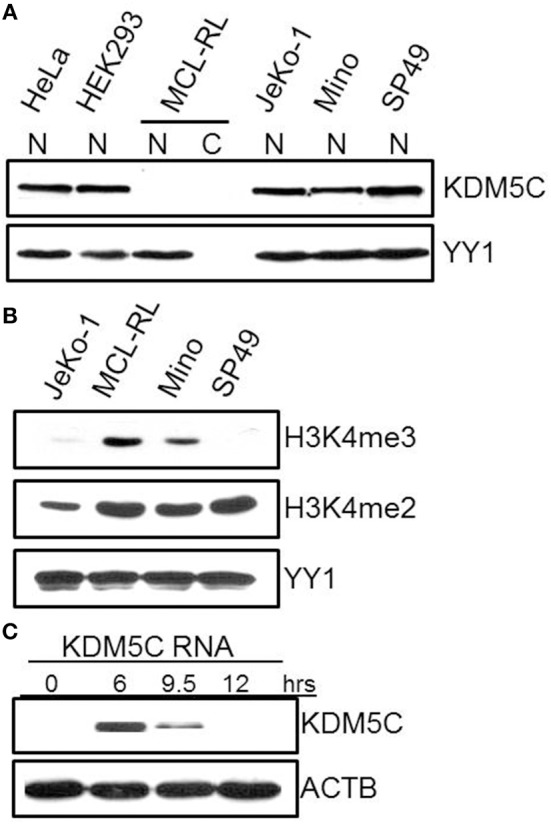
Expression of KDM5C protein and H3K4 methylation status in MCL-RL cells. **(A)** Evaluation of KDM5C expression in protein cell lysates of nuclear (N) and cytoplasmic (C) fractions from MCL-RL and the other depicted cell lines, both MCL-derived (JeKo-1, Mino, and SP49) and non-lymphoid (HeLa and HEK293). Expression of the nuclear protein YY1 served as positive control. **(B)** Di (me2) and tri(me3)-methylation status in nuclei of MCL-RL and other depicted MCL cell lines with expression of YY1 serving as positive control. **(C)** Expression of KDM5C protein in MCL-RL cells injected with KDM5C mRNA.

Finally, we have focused on mutations of three genes with functional links to methylation of histone 3 on lysine 4 (H3K4): KMT2D-p.Q3717H, BCOR-p.K439M, and KDM5C-p.S41^*^. Because BCOR and KDM5C genes are located on chromosome X, monoallelic mutations are functionally biallelic in males, as was the case in this patient. Noteworthily, all three mutations are also novel in cancer and considered oncogenic with scores of 0.885, 0.989, and 1,000, respectively. While the first two were identified late in the disease, in the 2013 primary and cultured cells, KDM5C mutation was seen through the entire lymphoma history, including the samples examined in 2005, 2007, 2009, and 2013. KMT2D is an H3K4 methyltransferase promoting chromatin opening and transcriptional activation of the targeted genes. It is frequently mutated at a number of positions throughout the gene body in diverse lymphoid and non-lymphoid malignancies (4, 21) including MCL (3). BCOR (BCL-6 co-repressor) specifically inhibits gene expressions when recruited to promoter regions by sequence-specific DNA binding proteins such as BCL6 (22, 23). BCOR has been also shown to interact with specific class I and II histone deacetylases (HDACs) and is involved in the inhibition of H3K4/36 methylation (22, 23). Mutations of BCOR at various coding sites have been frequently seen in lymphoid and myeloid malignancies but not so far in MCL. In turn, KDM5C is a demethylase (24–28) able to remove methyl residues from H3K4 at the di (me2)- and tri (me3) state. Diverse KDM5C mutations have been reported in both T-and B-cell lymphoid malignancies but not MCL. As the mutation pS41^*^ identified by us results in a creation of a stop codon, we examined the expression of KDM5C protein in the MCL-RL cells. As shown in Figure 4, not only there was no detectable KDM5C protein in these cells (panel A), but they also displayed upregulation of H3K4 di- and, in particular, tri-methylation (panel B).

To investigate the impact of KDM5C protein loss on MCL-RL cells, we transfected these cells with mRNA-KDM5C to induce expression of the KDM5C protein and examined changes in gene expression on a global scale using RNA-Seq (Figure 4C, Supplemental Table 6). Expression of KDM5C protein was induced rapidly with the expression peak observed as early as 6 h after the transfection, followed by a steady decline of the expression at 9 and 12 h (Figure 4C). As shown in Supplemental Table 6, the re-expressed KDM5C changed the expression by at least two-fold of numerous genes, many of which are involved in the regulation of cell proliferation, adherence, movement, and invasiveness. These genes include MAPK7, DUSP6, IL-8, SPP1 (a cytokine that regulates expression of interferon-gamma and interleukin-12), and PIK3IP1 [a negative regulator of PI3K activity (29)], and MBD3 (involved in nucleosome remodeling activities). Two of the other modulated genes participate in RNA transcription and translation: NEB (plays a key role in pre-mRNA formation) and ZBP1 (RNA-binding factor that recruits target transcripts to cytoplasmic protein-RNA complexes). These results suggest that loss of KDM5C impacts the biology of MCL in diverse mechanisms including modulation of cell signaling pathways.

## Discussion

Despite impressive progress made and introduction of new targeted therapies resulting in markedly extended progression-free survival, in particular in younger patients, MCL still remains a non-curable disease (1, 2). Recent reports shed light on the mutational makeup of the lymphoma (3–6). However, these studies identified only a few relatively frequent mutations with others being quite rare, suggesting substantial disease heterogeneity, and leaving the fundamental questions regarding the mechanisms of MCL development and progression still largely unanswered. Our longitudinal evaluation of mutational landscape in four samples and a cell line derived from the same MCL patient identified a large number of mutations seen only in malignant cells but not in the patient's normal cells as well as databases of benign SNPs. While some of the affected genes such as ATM, CCND1, and KMT2D are known to be mutated in MCL (3–6), a large subset has not been reported as mutated before in this lymphoma but mutated in other types of lymphoma, as detailed in the Results section. Some of the specific point mutations we have found have not been identified so far in cancer cells of any kind, although other mutations of the affected genes have been described. These novel mutations are deemed oncogenic, as predicted by the cutting-edge algorithms. In sum, our results derived from the analysis of multiple biopsies from a single patient further expand the list of mutations seemingly contributing to the pathogenesis of MCL and other malignancies. Remarkably, the core of 21 tumor-specific mutations (Figure 1 and Supplemental Table 1) remained preserved by the MCL lymphoma including the cell line in the span of at least 8 years, despite substantial fluctuation of mutations in other gene sets. This observation strongly suggests that at least some of these core mutations play an important role in the MCL pathogenesis with the caveat that this is the case in only a subset of patients, given the overall low to moderate frequency of these mutations in this type of lymphoma. The presence of this core group of mutated genes ATM and NEK1, both involved in DNA repair, suggests that derailment of the repair is one of such required pathogenic events in MCL.

Whereas, the IgH-CCND1 gene translocation leading to ectopic expression of CCND1 gene is a hallmark of MCL present in the vast majority of cases (1, 2), the significance of the “superimposed” CCND1 mutation also seen in all our samples remains undefined. The “hot spot” clustering of the mutations in exon 1 of the CCND1 gene (3) as well as our algorithmic analysis strongly suggest the oncogenic nature of the mutation. It is noteworthy that only the mutated form of CCND1 is expressed (Figure 3A), indicating that the gene mutation must have occurred essentially simultaneously with its translocation to the IgH promoter region and further supporting the notion of pathogenic relevance of the mutation. Our identification of mutations in as many as three genes involved in H3K4 methylation indicates that disruption of this process plays an important role in MCL, similar to other lymphomas (30). While the mutation-driven loss of KDM5C demethylase occurred early in the disease, mutations of KMT2D methyltransferase and BCOR factor, both also considered pathogenic, occurred late in the disease, as they were seen in only the last sample and the cell line. This order of events suggests that cessation of H3K4 demethylation was an initiating event tipping the balance toward increased H3K4 di-and tri-methylation and that the imbalance was further deepened in the advanced disease by the mutations of KMT2D and BCOR. Whereas, in diffuse large B-cell lymphoma a decrease, rather than increase in H3K4 methylation, seems pathogenic (30), KDM5C-driven augmentation of H3K4 methylation may contribute to the pathogenesis of acute B-cell leukemia (31); this is an observation similar to ours in MCL (Figure 4).

While the mutational analysis performed so far provided insights into MCL pathogenesis, a number of questions remain unanswered. While persistent BCR-BTK signaling is critical in this and other types of lymphoma, we and others (3–6) did not see any mutations potentially explaining the reliance of MCL on this signaling pathway. Our MCL-RL cell line should be a very valuable tool in this context, as it is highly sensitive to BTK inhibitors, a feature unique compared to standard MCL cell lines (Figure 2). These cells also are sensitive to inhibition of CDK4/6 and mTOR, two other kinases that play a role in MCL pathogenesis and therapy permitting evaluation of potentially the most effective drug combination in the *in vitro* and *in vivo* preclinical models. Of note, our data suggest that a combination of BTK inhibition with inhibition of either of the two other kinases is very beneficial but only in the presence of functional sensitivity to BTK inhibition (Figure 2). We also show that the MCL-RL cell line displays and maintains the key features of the patient-derived, primary malignant cells, including cytokine/receptor secretion patterns and genomic and epigenomic profiles. This comparative analysis, novel and most comprehensive for cultured and primary cancer cells of any kind derived from the same patient, indicate that MCL-RL cells are highly representative of the primary malignant cells and, hence, further validate this cell line as an experimental model of MCL.

In summary, we have shown that the mutational landscape of MCL is quite dynamic with dominant distinct sub-clones emerging and subsiding, most likely in response to the applied therapies. It is more than likely that the specific mutational landscape and, in particular, its changes are quite diverse in individual patients. However, the set of core mutations persisted in our patient for over a decade in all four primary samples examined as well the derived cell line affecting genes involved in DNA repair, cell cycle progression, and protein modifications. Among these persistent mutations, there was a nonsense mutation of H3K4 demethylase KDM5C accompanied at the late stage of the disease by missense mutations of KMT2D methyltransferase and BCOR, both implicated in H3K4 methylation, and associated with hypermethylation of H3K4 at me2 and me3. Future studies focused on the exact impact of mutations in H3K4 modifiers are needed to clearly understand their pathogenic role in MCL as well as other types of lymphoma and cancer at large.

Finally, our findings indicate that studying multiple biopsies from the same patients at various stages of the disease may facilitate identification of the core gene mutations responsible for the malignant cell transformation. On the clinical level, comprehensive genomic profiling of MCL biopsies seems warranted, given the marked mutational heterogeneity seen in this malignant disorder [(3–7) and this report].

## Author Contributions

QZ and MW designed the research. QZ, HW, XL, MHR, S-CL, Q-BX, MR, and HS performed experiments. SS took care of the patient, provided clinical history, facilitated obtaining the patient's primary MCL cells, and edited the manuscript. QZ, HW, MHR, AS, KJ, CS, AP, and MW analyzed the data. JG critically reviewed and revised the manuscript. AP and MW wrote the manuscript.

### Conflict of Interest Statement

The authors declare that the research was conducted in the absence of any commercial or financial relationships that could be construed as a potential conflict of interest.
