# Supplementary material for: Dynamic Changes in Gene Mutational Landscape With Preservation of Core Mutations in Mantle Cell Lymphoma Cells
Source: Front Oncol. 2019 Jul 3;9:568. doi: 10.3389/fonc.2019.00568 (PMC6617136; doi:10.3389/fonc.2019.00568)
Supplement: Supplementary file 1 [file Table_1.pdf]

Suppl. Table I. Comparative analysis of malignancy-specific mutations with high allelic burden in primary and cultured cells of MCL

Position	Gene symbol	AA Change	Ref. base	Genotype Call					Mutant frequency									
				Norm	2003	2005	2007	2013	Norm	2005	2007	2009	2013	C1-S*	C2-S	C1-L#	C2-L	C-RNA^
chrX.39933283	BCOR	K439M	T	TT	TT	TT	TT	AA	0	0.00	0.00	0.00	0.98	1	1	1	1	1.00
chrX.15554526	BMX	P400A	C	CC	CC	CC	CC	CG	0	0.00	0.00	0.00	0.92	1	1	1	1	-
chr9.77411762	TRPM6	F757L	A	AA	AA	AA	AA	AC	0	0.00	0.00	0.00	0.91	1	1	1	1	-
chr1.52698926	ZFYVE9	L16F	A	AA	AA	AA	AA	AC	0	0.00	0.00	0.00	0.73	0.71	0.62	0.81	0.79	0.62
chr1.43778125	TIE1	R594W	C	CC	CC	CC	CC	CT	0	0.00	0.00	0.00	0.70	0.80	0.67	0.8	0.78	-
chr7.107566759	LAMB1	S1645T	A	AA	AT	AT	AT	AT	0	0.31	0.45	0.33	0.67	0.59	0.63	0.63	0.55	1.00
chr6.75912505	COL12A1	R2W	G	GG	GG	GG	GG	GA	0	0.00	0.00	0.00	0.66	0.65	0.74	0.64	0.64	-
chr11.69456221	CCND1	C47S	G	GG	GC	GC	GC	GC	0	0.56	0.63	0.55	0.65	0.66	0.64	0.69	0.65	0.99
chr11.108224516	ATM	I2899L	A	AA	AC	AC	AC	AC	0	0.46	0.36	0.37	0.62	0.75	0.68	0.68	0.61	0.74
chr1.151681719	CELF3	R81Q	C	CC	CT	CT	CT	CT	0	0.39	0.53	0.39	0.62	0.75	0.73	0.79	0.78	-
chr6.30570667	PPP1R10	K638R	T	TT	TT	TT	TT	TC	0	0.00	0.00	0.00	0.58	0.72	0.63	0.69	0.67	1.00
chr6.121577274	TBC1D32	I631V	T	TT	TT	TT	TT	TC	0	0.00	0.00	0.00	0.55	0.6	0.75	0.58	0.62	1.0
chr11.1502012	MOB2	K72Q	T	TT	TT	TT	TT	TG	0	0.00	0.00	0.00	0.53	0.5	0.4	0.53	0.5	0.58
chr17.36830504	C17orf96	W82L	C	CC	CC	CC	CC	CA	0	0.00	0.00	0.00	0.52	0.45	0.47	0.57	0.48	-
chr2.120709686	PTPN4	M598I	G	GG	GT	GT	GT	GT	0	0.36	0.45	0.36	0.52	0.6	0.73	0.48	0.39	0.56
chr17.1628920	WDR81	A223T	G	GG	GG	GG	GG	GA	0	0.00	0.00	0.00	0.52	0.59	0.52	0.5	0.53	0.61
chr14.24884437	NYNRIN	R1161H	G	GG	GG	GG	GA	GA	0	0.00	0.00	0.34	0.5	0.46	0.43	0.52	0.49	0.75
chr4.153690722	TIGD4	L479V	A	AA	AA	AA	AA	AC	0	0.00	0.00	0.00	0.49	0.35	0.49	0.45	0.48	-
chr20.33509378	ACSS2	A494V	C	CC	CT	CT	CT	CT	0	0.36	0.46	0.37	0.49	0.55	0.43	0.45	0.49	0.36
chr16.68025054	DPEP2	F232L	G	GG	GG	GG	GG	GT	0	0.00	0.00	0.00	0.48	0.49	0.52	0.41	0.53	-
chr5.5306794	ADAMTS16	A1122S	G	GG	GT	GT	GT	GT	0	0.40	0.67	0.42	0.48	0.46	0.48	0.6	0.52	-
chr3.14536446	GRIP2	Q1055H	C	CC	CC	CC	CC	CG	0	0.00	0.00	0.00	0.48	0.45	0.53	0.61	0.44	-
chr3.142443557	TRPC1	L52F	G	GG	GG	GG	GG	GC	0	0.00	0.00	0.00	0.47	0.54	0.43	0.44	0.47	0.60
chr15.85607652	PDE8A	L80I	C	CC	CC	CC	CC	CA	0	0.00	0.00	0.00	0.47	0.6	0.59	0.57	0.62	0.25
chr3.69057600	EOGT	C97S	C	CC	CC	CC	CC	CG	0	0.00	0.00	0.00	0.47	0.45	0.45	0.44	0.41	0.50
chr5.148610254	ABLIM3	A267V	C	CC	CC	CC	CC	CT	0	0.00	0.00	0.00	0.47	0.47	0.49	0.46	0.45	-
chr4.170398451	NEK1	D753G	T	TT	TC	TC	TC	TC	0	0.48	0.44	0.52	0.46	0.53	0.46	0.48	0.46	0.60
chr7.117250620	CFTR	Q1012H	A	AA	AA	AA	AA	AT	0	0.00	0.00	0.00	0.46	0.55	0.6	0.53	0.56	-
chr7.7635993	MIOS	T768S	A	AA	AA	AA	AA	AT	0	0.00	0.00	0.00	0.46	0.38	0.37	0.37	0.41	0.35
chr12.88505575	CEP290	V705L	C	CC	CC	CC	CC	CG	0	0.00	0.00	0.00	0.46	0.51	0.42	0.45	0.42	0.35

Position	Gene symbol	Amino Change	Ref. base	Genotype Call					Mutant frequency									
				Norm	2003	2005	2007	2013	Norm	2003	2005	2007	2013	C1-S	C2-S	C1-L	C2-L	C-RNA
chr8.27880880	NUGGC	L782P	A	AA	AA	AA	AA	AG	0.00	0.00	0.00	0.00	0.45	0.47	0.49	0.46	0.46	0.53
chr2.139428811	NXPH2	S159I	C	CC	CA	CA	CA	CA	0.00	0.35	0.44	0.37	0.45	0.61	0.66	0.51	0.41	-
chr17.7460503	TNFSF12	S196T	T	TT	TT	TT	TT	TA	0.00	0.00	0.00	0.00	0.45	0.42	0.46	0.56	0.49	-
chr2.80801404	CTNNA2	V620I	G	GG	GA	GA	GA	GA	0.00	0.13	0.13	0.47	0.44	0.72	0.69	0.46	0.48	0.35
chr4.83825932	THAP9	V42L	G	GG	GC	GC	GC	GC	0.00	0.31	0.36	0.44	0.44	0.41	0.44	0.45	0.46	0.35
chr8.65517363	CYP7B1	R370H	C	CC	CC	CT	CT	CT	0.00	0.00	0.26	0.39	0.43	0.49	0.52	0.42	0.44	-
chr8.110476649	PKHD1L1	D2530H	G	GG	GG	GG	GG	GC	0.00	0.00	0.00	0.00	0.43	0.57	0.36	0.58	0.49	-
chr2.105472137	POU3F3	A57T	G	GG	GG	GG	GG	GA	0.00	0.00	0.00	0.00	0.41	0.5	0.62	0.39	0.68	-
chr3.42739110	HHATL	G252D	C	CC	CT	CT	CT	CT	0.00	0.37	0.28	0.40	0.41	0.5	0.45	0.49	0.54	-
chr18.44173689	LOXHD1	D435E	G	GG	GG	GG	GG	GT	0.00	0.00	0.00	0.00	0.4	0.46	0.53	0.42	0.37	-
chr9.134019784	NUP214	S471C	C	CC	CC	CC	CC	CG	0.00	0.00	0.00	0.00	0.54	0.58	0.63	0.53	0.52	0.41
chr6.118588218	SLC35F1	R180W	C	CC	CT	CT	CT	CT	0.00	0.38	0.28	0.43	0.65	0.63	0.56	0.72	0.61	-
chr8.94807677	TMEM67	A491V	C	CC	CT	CT	CT	CT	0.00	0.33	0.31	0.36	0.44	0.51	0.38	0.42	0.54	0.67
chr17.2236376	TSR1	K395T	T	TT	TT	TT	TT	TG	0.01	0.00	0.00	0.00	0.45	0.55	0.51	0.4	0.51	0.49
chr17.17900813	LRRC48	F288L	T	TT	TT	TT	TT	TG	0.01	0.00	0.00	0.00	0.5	0.57	0.44	0.46	0.54	-
chr12.49427337	KMT2D	Q3717H	C	CC	CC	CC	CC	CG	0.01	0.00	0.00	0.00	0.53	0.49	0.49	0.51	0.44	0.50
chr8.110255324	NUDCD1	I527V	T	TT	TT	TT	TT	TC	0.01	0.00	0.00	0.00	0.44	0.47	0.6	0.44	0.45	0.57
chr7.2983988	CARD11	Y181S	T	TT	TT	TT	TT	TG	0.01	0.00	0.00	0.00	0.43	0.45	0.46	0.4	0.4	0.48
chr1.71544230	ZRANB2	K43T	T	TT	TG	TG	TG	TG	0.01	0.31	0.25	0.42	0.59	0.66	0.78	0.66	0.73	0.86
chr10.92678714	ANKRD1	V121M	C	CC	CC	CC	CC	CT	0.01	0.00	0.00	0.00	0.5	0.53	0.53	0.45	0.49	-
chr14.75230812	YLPM1	Y207S	A	AA	AA	AA	AA	AC	0.01	0.00	0.00	0.00	0.45	0.48	0.48	0.51	0.47	0.50
chrX.50377589	SHROOM4	P495R	G	GG	GG	GG	GG	CC	0.01	0.00	0.00	0.00	0.96	1	1	1	1	-
chr21.35144452	ITSN1	R377H	G	GG	GA	GA	GA	GA	0.01	0.36	0.43	0.35	0.5	0.43	0.46	0.49	0.46	0.67
chr4.126367548	FAT4	T2432S	A	AA	AA	AA	AA	AT	0.02	0.00	0.00	0.00	0.45	0.5	0.56	0.49	0.43	-
chr8.87081757	PSKH2	A32V	G	GG	GG	GG	GG	GA	0.03	0.00	0.00	0.00	0.65	0.54	0.45	0.62	0.65	-
chr15.45003745	B2M	M1V	A	AA	AA	AA	AA	AG	0.00	0.00	0.00	0.00	0.84	1	1	1	1	0.98
chr13.26621004	SHISA2	R179C	G	GG	GG	GG	GG	GA	0.01	0.00	0.00	0.00	0.52	0.57	0.53	0.55	0.55	-
chr8.11058203	XKR6	A216S	C	CC	CC	CC	CC	CA	0.00	0.00	0.00	0.00	0.52	0.5	0.46	0.48	0.52	-
chr5.177546676	N4BP3	A31V	C	CC	CC	CC	CC	CT	0.00	0.00	0.00	0.00	0.48	0.45	0.46	0.49	0.46	0.72
chr18.44773552	SKOR2	H668P	T	TT	TT	TT	TT	TG	0.00	0.06	0.18	0.08	0.44	0.24	0.45	0.27	0.42	-
chr15.98509178	ARRDC4	R143L	G	GG	GT	GT	GT	GT	0.01	0.38	0.29	0.43	0.40	0.5	0.55	0.49	0.46	0.50

Position	Gene symbol	Amino Change	Ref. base	Genotype Call					Mutant frequency									
				Norm	2003	2005	2007	2013	Norm	2003	2005	2007	2013	C1-S	C2-S	C1-L	C2-L	C-RNA
chrX.53253950	KDM5C	S41X	G	GG	GT	GT	TT	TT	0.00	0.77	0.89	0.94	0.94	1.00	1.00	1.00	1.00	1.0
chr15.29996453	TJP1	K1709X	T	TT	TT	TT	TT	TA	0.00	0.00	0.00	0.00	0.86	1.00	1.00	1.00	1.00	-
chr11.74904432	SLCO2B1	C271X	C	CC	CA	CA	CA	CA	0.00	0.40	0.49	0.40	0.66	0.75	0.69	0.69	0.66	-
chr17.71282459	CDC42EP4	D61N	C	CT	CT	CT	CT	TT	0.43	0.45	0.49	0.55	0.97	1	1	1	1	-
chr9.4662692	PPAPDC2	L106R	T	TG	TG	TG	TG	TG	0.46	0.53	0.58	0.49	0.91	1	1	1	1	1.00
chr17.67171612	ABCA10	F938V	A	AC	AC	AC	AC	CC	0.47	0.48	0.47	0.36	0.95	1	1	1	1	-
chr17.27943049	CORO6	L403I	G	GT	GT	GT	GT	TT	0.47	0.50	0.44	0.47	1	1	1	1	1	-
chr17.66902228	ABCA8	E745D	T	TG	TG	TG	TG	GG	0.48	0.47	0.63	0.31	0.98	0.99	1	1	1	-

* CS-1 (and 2); MCL-RL cell line sub-lines 1 and 2 cultured for short time (3 months) prior to the analysis

CL-1 (and 2); the MCL-RL cell line sub-lines 1 and 2 cultured for longer period of time (6 months) prior to the analysis

^ C-RNA: RNA expression by the MCL-RL cell line of the mutated gene
