# Supplementary material for: Dynamic Changes in Gene Mutational Landscape With Preservation of Core Mutations in Mantle Cell Lymphoma Cells
Source: Front Oncol. 2019 Jul 3;9:568. doi: 10.3389/fonc.2019.00568 (PMC6617136; doi:10.3389/fonc.2019.00568)
Supplement: Supplementary file 2 [file Table_2.pdf]

Supplemental Table II. Cytokine and soluble cytokine receptor expression by primary and cultured MCL-RL

Name	EGF	Eotaxin	FGF-Basic	G-CSF	GM-CSF	HGF	IFN-a	IFN-g	IL-10	IL-12	IL-13	IL-15	IL-17	IL-1B	IL-1RA
(SC) Max (pg/ml)	2133.11	3438.95	1153.58	58843.35	14150.11	13097.27	4376.34	10697.69	20366.43	11307.08	5967.44	28027.28	22640.84	8757.39	22073.21
(SC) Min (pg/ml)	1.11	0.56	0.54	9.11	2.71	1.78	1.93	1.76	3.37	1.77	3.15	11.61	3.35	1.34	10.49
Luminex reference	15.40	1.02	15.71	278.85	766.71	205.37	112.06	3158.59	21.60	77.61	99.73	245.68	9.06	205.34	2276.24
JeKo-1 SUPERNATANT	2.46	0.74	0.00	54.82	1.59	57.66	12.11	7.19	2.52	25.32	26.27	103.15	0.00	9.91	0.00
MINO SUPERNATANT	15.90	0.90	12.92	90.18	1.59	29.68	30.59	8.34	4.45	47.22	28.08	175.11	0.00	19.48	0.00
SP49 SUPERNATANT	18.11	0.97	12.66	96.84	1.87	36.29	29.17	4.16	164.81	32.62	16.88	203.77	0.00	21.88	0.00
**MCL-RL SUPERNATANT**	0.00	87.51	0.00	255.05	2.92	3753.71	101.80	8.32	34.22	417.30	56.54	168.02	0.00	0.00	346.20
**MCL- RL PLEURAL FLUID**	0.00	2.03	0.00	104.42	2.06	121.37	35.25	10.51	28.47	83.76	28.29	171.34	0.00	0.00	18.10

Name	IL-2	IL-2Ra	IL-4	IL-5	IL-6	IL-7	IL-8	IP-10	MCP-1	MIG	MIP-1a	MIP-1b	RANTES	TNF-a	VEGF
(SC) Max (pg/ml)	10755.60	23191.09	40862.96	5075.61	4603.70	6610.26	10207.32	4111.01	14958.18	3876.26	6553.53	8265.08	3131.23	6753.79	4654.89
(SC) Min (pg/ml)	1.76	3.51	6.59	0.78	0.76	0.74	1.60	0.67	2.21	0.67	2.93	1.26	1.72	1.06	0.93
Luminex reference	20433.87	556.82	41.73	83.43	983.79	38.31	38585.32	555.75	4214.85	1643.97	6553.53	9221.55	711.16	804.95	101.29
JeKo-1 SUPERNATANT	0.00	108.46	3.36	0.37	16.66	35.29	9.77	4.28	23.72	4.79	648.60	203.16	178.60	2.04	150.20
MINO SUPERNATANT	2.26	266.01	4.38	0.50	16.79	39.75	13.93	9.26	28.81	3.98	17306.04	>8265.08	638.66	5.56	128.88
SP49 SUPERNATANT	2.60	291.98	4.59	0.34	9.61	27.59	42.00	484.39	53.84	29.61	>6553.53	>8265.08	185.66	6.84	61.75
**MCL-RL SUPERNATANT**	0.00	2314.20	18.63	26.63	>4603.70	55.44	19367.93	2334.35	4618.27	25.26	841.79	1428.88	144.05	40.79	211.00
**MCL-RL PLEURAL FLUID**	0.00	803.33	6.30	1.39	2191.38	32.05	825.18	4985.17	230.45	32.87	>6553.53	18997.72	639.20	84.60	96.18

MCL-RL unique		**Eotaxin***	FGF											IL-18	
common with the other MCL cell lines	EGF[#]			**G-CSF**	GM-CSF	**HGF**	**IFN-a**	IFN-g	**IL-10**	**IL-12**	**IL-13**	**IL-15**	IL-17		**IL-1RA**
MCL-RL unique		**IL-2Ra**	IL-4	**IL-5**	**IL-6**		**IL-8**							**TNFa**	
common with the other MCL cell lines	IL-2					IL-7		**IP-10**	**MCP-1**	**MIG**	**MIP-1a**	**MIP-1b**	**RANTES**		**VEGF**

*strong expression (in bold)

low/lack of expression (regular font)
