# Supplementary material for: Dynamic Changes in Gene Mutational Landscape With Preservation of Core Mutations in Mantle Cell Lymphoma Cells
Source: Front Oncol. 2019 Jul 3;9:568. doi: 10.3389/fonc.2019.00568 (PMC6617136; doi:10.3389/fonc.2019.00568)
Supplement: Supplementary file 3 [file Table_3.pdf]

Supplemental Table III. CpG site methylation in primary and cultured MCL cells.

		# of CpG sites with changed methylation			
	Read depth >=10	>=50% methylation change		>=70% methylation change	
	Total site number	Increase	Decrease	Increase	Decrease
RL-P==>RL1-C1*	2186253	3671	6587	234	481
RL-P==>RL1-C2	1901699	3872	7411	246	623
RL-P==>RL2-C1	2245753	4352	8048	304	649
RL-P==>RL2-C2	2283968	5838	9618	437	931

*Abbreviations: RL-P; patient's primary MCL cells, RL1 and RL2; sub-lines of MCL-RL cell line, C1 and C2; cells cultured for 3 and 5 months, respectively.
