# Supplementary material for: Dynamic Changes in Gene Mutational Landscape With Preservation of Core Mutations in Mantle Cell Lymphoma Cells
Source: Front Oncol. 2019 Jul 3;9:568. doi: 10.3389/fonc.2019.00568 (PMC6617136; doi:10.3389/fonc.2019.00568)
Supplement: Supplementary file 4 [file Table_4.pdf]

Supplemental Table IV. Promoter region methylation pattern in primary and cultured MCL cells (page 1/7).

Symbol	IsLand Length	CpG Count	CpGs detected	Methylation Ratio (%)					Change from Primary to	
				RL-P*	RL1-C1	RL1-C2	RL2-C1	RL2-C2	RL1-C2	RL2-C2
YBX1P4	750	65	22	98.23	98.24	97.63	98.06	96.36	-0.60	-1.87
RGPD1	321	36	18	97.87	95.76	96.49	97.74	97.58	-1.38	-0.29
RMI2	489	42	12	97.60	97.90	94.69	97.62	96.97	-2.91	-0.63
KCNG2	1015	114	29	97.57	97.18	98.02	96.03	98.38	0.45	0.81
AP005901.1	626	47	11	97.32	96.31	98.18	98.39	98.93	0.86	1.61
BMPR1APS2	253	40	15	96.96	96.37	96.26	95.07	96.62	-0.70	-0.34
ZNF540	834	66	37	96.83	97.72	96.77	98.51	98.14	-0.06	1.30
RP11-206P5.2	1495	132	12	96.64	96.88	99.36	97.96	97.17	2.72	0.52
RP11-174O3.1	826	71	10	96.45	95.61	97.69	98.58	98.00	1.24	1.55
LINC00200	297	34	11	96.39	94.79	97.20	94.64	98.47	0.81	2.08
TDRD9	705	84	17	96.24	96.58	96.35	96.81	94.76	0.11	-1.48
RP11-666A20.3	1281	97	15	96.05	94.68	94.99	92.58	93.79	-1.05	-2.26
EXOC3L2	273	23	15	95.99	89.28	91.79	96.67	96.65	-4.20	0.66
CACNA1B	2213	216	30	95.65	95.48	95.30	94.78	95.66	-0.34	0.02
RP11-286H14.4	1647	122	25	95.58	96.28	96.42	96.50	97.14	0.83	1.56
AC008267.1	414	50	21	95.34	92.29	95.06	95.40	93.04	-0.28	-2.30
RP11-496I2.6	2265	209	49	94.71	94.79	93.58	93.30	93.40	-1.13	-1.32
CTD-3220F14.1	418	33	10	94.28	93.21	91.17	92.70	89.83	-3.11	-4.45
USP29	294	32	28	94.19	92.70	94.11	93.54	92.00	-0.08	-2.19
ZNF497	2248	227	80	94.14	94.18	94.21	95.01	94.94	0.07	0.81
LINC00221	622	57	34	94.11	93.64	92.63	94.69	93.95	-1.49	-0.16
IGHV2OR16-5	1036	89	23	93.57	93.98	94.42	91.07	91.29	0.85	-2.27
AC060834.2	554	44	14	92.99	88.60	93.74	89.72	90.85	0.74	-2.14
GRAMD4P6	620	50	10	92.95	92.92	91.51	91.75	87.72	-1.44	-5.23
LRRC72	422	34	15	92.81	87.51	89.00	90.91	90.59	-3.81	-2.22
NKX6-3	352	27	10	92.13	91.68	88.32	95.80	93.00	-3.81	0.87
RP11-165H4.3	1763	144	36	92.08	94.12	93.38	95.99	95.11	1.30	3.03
FBXO46	529	57	12	92.03	92.61	93.52	93.34	92.24	1.49	0.22
AC136297.1	909	70	12	91.33	90.83	91.74	95.82	92.88	0.42	1.56
RP11-736N17.9	570	79	14	91.28	92.67	96.75	95.71	96.35	5.47	5.07
DCDC2C	1099	125	17	91.24	96.00	96.92	95.16	95.95	5.69	4.72
CETN1	720	52	20	90.67	82.91	81.34	78.93	76.26	-9.33	-14.41
HOXA3	339	24	11	90.57	87.18	84.08	88.01	87.45	-6.49	-3.13
OR2L13	401	38	16	90.51	91.78	93.31	91.81	91.61	2.80	1.10
RP11-700P18.1	290	40	12	90.18	88.33	86.67	81.04	75.38	-3.51	-14.80

Supplemental Table IV. Promoter region methylation pattern in primary and cultured MCL cells (page 2/7).

Symbol	IsLand Length	CpG Count	CpGs detected	Methylation Ratio (%)					Change from Primary to	
				RL-P	RL1-C1	RL1-C2	RL2-C1	RL2-C2	RL1-C2	RL2-C2
Z95704.3	752	89	18	90.13	95.41	92.53	95.42	93.52	2.40	3.38
AC074389.5	725	59	46	90.12	88.97	88.17	89.80	90.88	-1.95	0.77
ACTN2	851	84	15	90.00	97.05	96.00	92.81	91.51	6.00	1.51
SEC23B	410	29	11	89.47	88.05	89.38	88.37	87.04	-0.09	-2.44
RP11-460N20.4	378	36	10	88.97	86.60	89.23	89.24	89.65	0.26	0.68
DUX4L8	27227	2005	84	88.97	91.42	90.85	90.38	90.75	1.89	1.78
MKRN4P	486	50	11	88.95	85.50	84.84	85.56	89.16	-4.11	0.22
RP11-60C6.6	798	85	65	88.94	94.55	93.08	92.22	92.10	4.14	3.16
NRN1L	230	18	14	88.88	89.43	89.53	85.77	87.25	0.65	-1.63
FZD9	2099	206	101	88.75	93.01	90.97	92.15	91.18	2.22	2.43
EXOC3L2	663	87	40	88.63	91.66	88.51	89.56	92.41	-0.13	3.78
MIR3675	412	41	11	88.41	88.95	87.17	88.00	89.45	-1.24	1.05
MIR3147	481	38	16	88.31	94.11	97.07	95.86	92.04	8.76	3.72
SNORA31	798	59	21	88.03	86.57	88.28	88.41	85.22	0.25	-2.80
ZSCAN1	782	98	12	87.38	93.09	93.32	91.20	97.50	5.93	10.12
AL355149.1	412	41	21	86.77	92.87	87.00	87.44	87.47	0.23	0.70
ZNF876P	515	49	37	86.14	87.83	89.77	89.87	89.94	3.62	3.79
LEP	625	60	11	86.10	89.68	89.87	89.83	94.54	3.77	8.44
ESPNP	400	38	15	85.57	85.04	86.25	84.53	83.09	0.68	-2.47
HOXD8	2078	186	12	84.97	96.15	91.66	94.82	96.43	6.69	11.46
RP11-113D6.10	287	29	13	84.87	95.12	97.08	94.47	94.02	12.22	9.15
C19orf77	802	66	17	84.56	87.40	89.11	81.26	82.69	4.55	-1.88
PIP5K1P2	304	26	13	84.36	78.97	81.38	78.29	74.06	-2.98	-10.30
AFG3L2P1	433	50	15	84.11	87.01	88.47	89.11	87.65	4.35	3.54
ZNF578	384	47	11	84.07	83.22	85.74	87.96	93.24	1.66	9.16
DSG2	974	80	76	84.02	84.00	85.85	87.86	88.22	1.82	4.20
HTR5BP	1588	155	15	83.89	91.41	92.20	86.88	87.21	8.31	3.32
RASGRF2	1102	131	10	83.74	91.47	95.54	85.10	93.15	11.80	9.41
NFE2L3P1	458	59	47	83.58	86.19	89.57	85.69	84.83	5.99	1.25
RP11-417J8.3	487	46	11	83.12	83.37	81.38	82.71	79.90	-1.74	-3.22
LOXHD1	213	20	19	82.98	79.32	77.85	81.91	83.72	-5.14	0.74
POM121L12	526	40	23	82.33	82.32	78.68	83.48	84.65	-3.65	2.33
SALL3	4239	338	162	82.23	85.05	86.82	84.08	85.35	4.59	3.11
RP4-725G10.4	892	94	14	81.07	84.09	81.77	80.85	81.10	0.70	0.03
SYT14	875	99	18	80.91	79.73	81.71	84.17	83.59	0.79	2.68

Supplemental Table IV. Promoter region methylation pattern in primary and cultured MCL cells (page 3/7).

Symbol	IsLand Length	CpG Count	CpGs detected	Methylation Ratio (%)					Change from Primary to	
				RL-P	RL1-C1	RL1-C2	RL2-C1	RL2-C2	RL1-C2	RL2-C2
FZD6	811	84	18	80.65	88.42	91.56	87.02	89.81	10.91	9.16
PCDHB10	902	84	12	80.39	78.91	82.81	81.92	81.81	2.42	1.42
RBM46	988	92	13	80.35	82.66	71.86	80.37	75.08	-8.48	-5.27
KRTAP5-6	241	18	14	80.30	73.69	77.36	75.34	70.24	-2.94	-10.06
UPF3A	1280	135	13	80.03	72.20	69.64	71.65	80.15	-10.39	0.12
DLK1	648	65	21	79.80	84.80	82.08	86.44	88.69	2.27	8.88
POU4F3	1806	138	27	79.64	85.26	88.71	83.73	83.18	9.08	3.54
GUSBP6	645	63	13	79.15	81.12	75.88	66.52	73.55	-3.26	-5.59
LPPR1	1152	98	11	79.07	90.33	80.11	75.73	75.84	1.04	-3.24
RP11-764K9.1	667	53	16	78.62	73.19	80.96	76.76	73.05	2.34	-5.57
AF186192.6	1216	118	21	78.61	83.47	79.32	79.75	79.11	0.71	0.50
ASPG	466	53	43	78.57	83.19	82.37	82.12	86.17	3.80	7.60
EVX1	1050	81	20	78.55	79.94	81.66	78.51	77.27	3.11	-1.28
SLC6A11	760	87	11	78.53	63.37	76.40	77.73	66.81	-2.13	-11.72
RP11-715L17.1	1564	139	56	78.51	76.06	78.09	77.76	77.69	-0.42	-0.82
HDAC9	1095	105	55	78.42	77.71	77.98	74.32	79.22	-0.44	0.80
FEZF2	472	51	24	78.05	77.93	79.66	82.97	82.02	1.61	3.97
VSTM2A	406	39	34	77.81	78.68	81.65	81.39	80.66	3.84	2.85
TMEM132C	2110	184	13	77.05	80.69	78.17	72.18	80.65	1.12	3.59
CYP26C1	3226	255	17	76.71	79.61	85.96	86.08	86.38	9.26	9.67
CWH43	1018	94	20	76.67	84.33	81.83	77.04	75.99	5.16	-0.67
RBM24	1595	135	12	76.45	76.70	78.77	80.12	77.09	2.32	0.64
TUBBP5	605	61	12	76.30	68.35	74.79	64.78	65.11	-1.51	-11.19
HRASLS	664	84	10	76.18	83.35	79.50	82.21	82.18	3.32	6.00
FAAH	567	71	13	75.70	84.61	78.08	89.08	83.20	2.38	7.50
TKTL1	434	32	16	75.58	76.78	74.59	74.86	75.78	-0.99	0.20
GLDN	594	69	24	75.46	74.78	80.40	81.78	79.68	4.94	4.22
MYH14	714	74	20	75.01	79.76	80.72	76.78	78.89	5.71	3.88
SSTR5	2171	167	28	74.91	71.72	74.20	72.73	77.62	-0.72	2.70
RP11-154H12.2	403	32	14	74.83	72.99	79.62	81.64	77.64	4.79	2.81
TMC7	421	45	19	74.57	71.32	72.73	78.13	77.32	-1.84	2.75
ANO1	859	106	50	73.95	83.27	82.47	82.02	83.21	8.53	9.26
RP11-603B24.2	2073	182	125	73.85	68.34	64.99	65.78	68.17	-8.86	-5.68
KCNS2	1733	178	10	73.77	78.88	76.91	78.18	81.27	3.14	7.50
KCNK3	948	95	26	73.70	86.46	88.34	83.88	87.25	14.64	13.55

Supplemental Table IV. Promoter region methylation pattern in primary and cultured MCL cells (page 4/7).

Symbol	IsLand Length	CpG Count	CpGs detected	Methylation Ratio (%)					Change from Primary to	
				RL-P	RL1-C1	RL1-C2	RL2-C1	RL2-C2	RL1-C2	RL2-C2
TMEM215	689	77	22	72.93	77.54	73.20	64.10	71.33	0.28	-1.60
PTPN3	307	36	22	72.91	74.15	73.74	71.27	78.70	0.83	5.79
MIR200B	2541	183	59	72.88	74.83	75.26	74.01	76.50	2.38	3.62
TBX4	3531	238	24	72.07	66.15	67.68	67.38	73.01	-4.39	0.95
NEFH	1323	181	22	71.96	77.27	76.17	71.43	77.18	4.21	5.22
EGFR	1964	192	55	71.84	75.10	74.68	73.70	73.99	2.84	2.15
AC006033.22	411	38	15	71.77	71.78	66.37	73.41	77.31	-5.41	5.53
FFAR1	757	56	13	71.70	78.09	77.95	75.62	74.41	6.25	2.71
LRRC37A7P	294	26	11	71.66	72.65	72.85	75.59	73.30	1.19	1.64
WI2-2373I1.2	2170	223	119	71.60	75.51	73.34	73.79	75.59	1.74	4.00
HHIPL1	786	84	36	71.34	81.44	75.07	75.84	71.77	3.73	0.43
ZNF595	474	46	58	71.09	65.73	66.12	67.06	67.03	-4.97	-4.06
SNTG1	590	49	10	71.00	68.00	63.61	80.53	76.39	-7.39	5.39
RP11-107C16.2	317	36	17	70.98	78.98	73.95	72.99	71.10	2.96	0.12
PLK5	860	116	24	70.56	76.62	77.01	75.58	81.62	6.45	11.06
TBX20	1297	111	35	70.42	74.57	72.47	70.84	67.18	2.06	-3.23
OSR1	687	60	27	70.33	73.16	76.63	76.47	76.86	6.30	6.53
HS3ST4	1676	134	14	69.80	31.81	44.73	40.01	24.64	-25.07	-45.16
TMEM181	1457	151	16	69.75	77.47	76.13	74.85	74.67	6.38	4.92
CTD-2194D22.2	1671	125	15	69.61	69.47	71.01	69.47	61.71	1.41	-7.90
SKOR2	452	45	26	69.23	76.82	75.37	67.50	74.90	6.13	5.67
VWC2	2744	251	105	69.07	72.05	70.08	73.71	74.12	1.01	5.06
EN2	5565	404	75	68.68	72.68	73.61	73.09	75.13	4.93	6.46
ADCY1	2118	151	88	68.44	72.49	72.96	72.24	70.72	4.53	2.28
SLC35F3	1159	126	22	68.30	73.00	71.21	74.47	72.30	2.92	4.01
TNFRSF18	310	25	14	68.24	57.74	60.15	70.02	67.51	-8.09	-0.73
RP11-129I19.2	2284	196	30	68.21	67.04	70.65	64.70	69.62	2.45	1.42
RP11-403B2.6	856	89	10	67.95	56.66	78.19	48.06	78.04	10.24	10.09
ZFP37	1345	119	18	67.94	71.22	74.17	71.88	72.69	6.23	4.76
AURKC	502	40	14	67.36	72.25	68.70	60.74	65.08	1.34	-2.28
ZAR1	1472	168	19	66.91	75.00	72.49	81.13	79.97	5.58	13.06
GALR1	2266	220	88	66.88	70.96	73.33	72.91	73.80	6.45	6.92
SLC22A31	373	39	40	66.81	70.70	72.39	72.88	79.08	5.59	12.28
RP11-209K10.2	915	87	42	66.62	71.21	74.00	69.84	77.08	7.38	10.46
SYT9	1478	139	44	66.50	75.87	79.50	75.76	79.11	12.99	12.60

Supplemental Table IV. Promoter region methylation pattern in primary and cultured MCL cells (page 5/7).

Symbol	IsLand Length	CpG Count	CpGs detected	Methylation Ratio (%)					Change from Primary to	
				RL-P	RL1-C1	RL1-C2	RL2-C1	RL2-C2	RL1-C2	RL2-C2
AMZ1	298	32	33	66.21	74.80	75.30	73.83	74.17	9.10	7.96
SLC35F1	1769	166	15	66.11	55.19	57.22	54.45	53.29	-8.89	-12.81
FAM110B	821	95	17	65.65	69.76	76.18	75.80	76.45	10.54	10.80
LINC00273	2061	207	33	64.70	66.15	68.64	69.59	69.45	3.94	4.75
TRIM58	922	101	30	64.08	57.40	78.12	36.12	57.46	14.04	-6.62
LPHN3	2918	212	13	64.06	61.65	60.04	61.15	66.19	-4.02	2.13
BHLHA9	1198	129	25	63.71	77.40	80.64	75.39	77.84	16.93	14.12
FAM84A	3432	244	79	63.67	68.26	70.55	69.04	74.48	6.88	10.81
SLC6A19	695	51	10	63.29	61.73	54.17	64.46	67.30	-9.12	4.01
COL2A1	204	17	10	62.89	70.20	64.25	75.00	73.69	1.36	10.80
RP13-895J2.7	757	74	25	62.69	74.11	75.60	70.45	72.63	12.92	9.94
DIO3	5578	407	197	62.67	64.10	65.83	65.04	67.91	3.16	5.24
RP11-379F12.4	6955	509	16	62.45	71.09	62.92	66.13	69.52	0.47	7.07
PAX1	1490	141	21	62.25	70.92	79.94	76.52	78.45	17.69	16.20
RP11-451H23.2	509	56	26	62.24	67.99	75.63	69.83	77.95	13.39	15.71
DUX4L16	1323	92	20	62.04	59.17	53.19	56.02	54.39	-8.85	-7.65
NRG1	1821	143	27	61.77	67.21	77.64	74.90	77.50	15.87	15.73
CIDEA	942	85	39	61.65	66.34	60.02	69.13	67.91	-1.64	6.26
AP000357.4	262	32	11	61.51	78.15	77.78	74.91	73.16	16.27	11.65
RP11-545A16.3	369	38	11	61.26	73.46	76.39	72.73	71.94	15.13	10.67
ZNF578	589	47	12	61.13	42.52	61.43	73.20	64.87	0.31	3.74
MEST	2372	177	19	61.01	58.56	65.55	69.93	65.96	4.54	4.95
HMX3	3291	300	30	60.96	65.47	64.64	68.46	73.53	3.68	12.57
NKX2-8	556	45	10	60.64	86.95	80.56	77.85	77.83	19.92	17.19
EMX2	4047	313	11	60.43	67.53	61.32	63.65	58.32	0.89	-2.11
MAGI2	902	70	25	60.24	57.74	70.86	64.45	69.51	10.62	9.28
CHST8	2074	232	30	60.00	70.47	69.16	66.18	70.36	9.16	10.36
TFAP2E	1124	118	38	59.79	50.27	59.31	57.26	62.61	-0.48	2.82
NR2F2	4313	287	11	59.40	62.11	62.68	62.98	58.22	3.28	-1.18
UNCX	13699	923	276	59.38	62.30	63.81	64.31	64.33	4.44	4.95
TRIM67	1965	209	22	59.24	65.82	65.28	68.07	57.22	6.04	-2.02
ABCC9	1403	90	11	58.85	58.11	57.70	58.25	70.11	-1.15	11.25
IFNL3	1599	137	48	57.73	62.44	62.89	60.72	62.61	5.16	4.88
SLC6A2	1297	120	24	57.65	59.86	63.18	55.66	58.35	5.53	0.70
NAV2	1384	111	18	57.63	56.56	62.34	60.57	62.84	4.72	5.21

Supplemental Table IV. Promoter region methylation pattern in primary and cultured MCL cells (page 6/7).

Symbol	IsLand Length	CpG Count	CpGs detected	Methylation Ratio (%)					Change from Primary to	
				RL-P	RL1-C1	RL1-C2	RL2-C1	RL2-C2	RL1-C2	RL2-C2
LINC00466	8077	561	76	57.49	60.21	61.16	61.96	62.26	3.67	4.77
ZNF845	207	17	15	57.49	53.17	52.55	56.54	49.93	-4.93	-7.56
DUSP5P1	40058	2691	173	57.19	56.95	55.85	54.87	55.88	-1.34	-1.31
WWC2	2549	245	12	57.08	64.24	59.99	64.64	63.41	2.91	6.33
FAM163A	2004	199	29	57.04	64.69	60.31	44.56	62.26	3.27	5.21
DPP6	2349	212	32	57.03	60.26	60.26	51.57	56.86	3.23	-0.17
NPTX2	1954	233	28	56.96	68.68	65.55	57.91	65.11	8.59	8.15
RNA5SP519	369	38	10	56.88	44.52	61.04	51.10	49.66	4.16	-7.22
ADCYAP1	4996	330	66	56.66	59.65	59.44	59.40	60.79	2.78	4.13
PABPC1P5	1324	104	58	56.52	55.52	55.56	55.98	56.59	-0.96	0.07
RNA5-8SP5	1963	187	46	56.38	55.00	60.40	61.77	67.47	4.02	11.08
AC009531.2	581	48	16	56.26	62.14	62.13	59.54	66.40	5.86	10.14
SOX9	1447	97	11	55.99	56.10	56.52	52.08	54.48	0.53	-1.51
MTNR1B	645	60	19	55.58	57.59	60.40	58.87	62.79	4.82	7.21
MLPH	1180	104	11	55.33	51.43	72.86	69.18	66.11	17.54	10.78
NANOS3	312	29	20	55.13	61.50	70.38	51.44	56.02	15.25	0.89
IFNL2	1567	135	60	54.96	59.59	58.41	53.98	56.73	3.45	1.77
MIR3648	854	120	29	54.95	54.88	54.66	58.20	53.14	-0.29	-1.81
CDH20	1009	101	33	54.81	52.88	61.36	57.30	60.65	6.55	5.84
IRX5	5383	453	13	54.73	63.29	57.79	60.91	61.57	3.06	6.84
SRRM3	474	52	11	54.53	55.83	52.41	52.24	54.51	-2.12	-0.02
NPY	1522	123	51	54.43	49.07	52.58	57.29	57.12	-1.85	2.69
IRX1	8587	584	90	53.83	57.45	61.99	57.74	61.55	8.16	7.72
XKR4	2105	172	25	53.28	57.09	62.63	54.49	60.91	9.35	7.63
SLC34A2	428	50	16	53.28	56.68	60.28	54.16	56.76	7.00	3.49
OSMR	974	100	12	53.04	61.78	62.50	58.42	57.92	9.46	4.88
FOXD2	724	54	18	52.93	56.47	62.92	54.25	63.58	9.98	10.65
PTGDR	1279	111	25	52.84	59.64	59.87	59.15	57.85	7.03	5.01
TDGF1	362	26	16	52.67	62.93	51.43	56.81	61.74	-1.24	9.08
CHODL	776	77	12	52.60	56.91	55.56	52.90	56.72	2.96	4.12
NHS	2658	194	13	52.53	56.03	54.69	58.39	59.59	2.16	7.06
RP11-1055B8.6	7936	634	106	52.48	56.09	54.92	51.13	57.85	2.44	5.37
CTB-158E9.2	389	29	11	52.44	60.05	62.51	62.48	61.72	10.07	9.28
CTC-523E23.5	458	41	13	52.43	38.20	47.25	36.55	34.73	-5.18	-17.70
SPDYA	660	81	32	52.38	49.31	53.87	55.68	58.74	1.49	6.37

Supplemental Table IV. Promoter region methylation pattern in primary and cultured MCL cells (page 7/7).

Symbol	IsLand Length	CpG Count	CpGs detected	Methylation Ratio (%)					Change from Primary to	
				RL-P	RL1-C1	RL1-C2	RL2-C1	RL2-C2	RL1-C2	RL2-C2
DPP6	2037	165	15	52.37	53.90	62.56	61.40	66.47	10.19	14.10
RP11-79P5.3	2301	193	32	52.28	48.74	49.16	51.44	52.30	-3.13	0.02
CDX2	407	42	24	52.15	52.28	53.03	57.25	65.25	0.88	13.10
SIM2	5698	438	76	52.08	54.27	57.49	52.81	53.61	5.42	1.53
PCP4L1	551	54	17	51.98	56.41	48.53	60.71	61.98	-3.45	10.00
NKX2-1	894	63	19	51.97	54.67	56.67	55.83	55.05	4.70	3.08
DPY19L2P4	1144	98	31	51.79	57.93	62.33	51.12	55.19	10.54	3.41
SOX14	1490	110	26	51.34	55.75	57.08	59.89	58.72	5.73	7.38
GATA4	3360	221	48	51.07	55.46	60.23	54.70	55.82	9.15	4.74
RP11-391M20.1	883	76	21	51.01	45.86	45.81	39.32	48.90	-5.20	-2.11
FBXL7	847	98	24	50.99	51.45	51.16	54.64	58.23	0.17	7.24
MYOD1	2990	233	27	50.88	52.47	53.40	56.82	53.67	2.52	2.79
GAD1	3137	246	17	50.85	52.50	53.79	52.41	53.82	2.94	2.97
FGF2	1240	127	52	50.78	44.05	49.69	48.17	47.34	-1.09	-3.44
HS3ST2	1843	187	42	50.53	61.05	58.22	69.27	66.38	7.69	15.85
AP001626.1	202	17	11	50.52	46.09	55.45	31.53	37.87	4.93	-12.65
CDH4	2001	230	38	50.09	45.33	47.04	48.99	51.01	-3.05	0.91
MSX1	559	41	12	50.08	53.28	58.14	53.14	58.08	8.07	8.00

*Abbreviations: RL-P; patient's primary MCL cells, RL1 and RL2; sub-lines of MCL-RL cell line, C1 and C2; cells cultured for 3 and 5 months, respectively.
