# Supplementary material for: Dynamic Changes in Gene Mutational Landscape With Preservation of Core Mutations in Mantle Cell Lymphoma Cells
Source: Front Oncol. 2019 Jul 3;9:568. doi: 10.3389/fonc.2019.00568 (PMC6617136; doi:10.3389/fonc.2019.00568)
Supplement: Supplementary file 6 [file Table_6.pdf]

Supplementary Table VI. List of genes with expression affected by KDM5C expression in MCL-RL cells (thresholds: expression fold change >=2 change and FPKM >=5.0)

Gene	Fold change		FPKM			
	6hrs	12hrs	Ctr-6hrs	Tre-6hrs	Ctr-12hrs	Tre-12hrs
KDM5C	46.70	18.34	10.53	16.07	10.29	14.49
GOLGB1	2.27	1.21	9.89	11.07	10.98	11.26
TTR	2.19	1.00	9.25	10.39	0.00	0.00
PDK4	10.91	1.07	5.89	9.34	1.10	1.19
LINC00158	2.19	1.37	7.69	8.82	7.73	8.18
SERPINA9	3.13	1.68	7.13	8.77	6.14	6.88
SST	3.18	1.00	6.73	8.40	0.00	0.00
IL8	5.86	1.43	5.72	8.27	4.73	5.25
CLU	3.88	1.37	6.27	8.22	1.48	1.94
GADD45B	2.02	1.24	7.04	8.06	7.08	7.39
NEB	2.14	1.09	6.91	8.01	7.88	8.00
SULF2	2.89	1.33	6.45	7.98	5.98	6.39
TTN	2.06	1.07	6.71	7.75	8.24	8.33
CCDC110	2.04	1.53	5.86	6.88	6.63	7.24
REG1A	3.77	1.00	4.88	6.80	0.00	0.00
PCSK1	2.36	1.00	5.27	6.51	0.00	0.00
LOC348840	2.78	2.30	4.98	6.45	3.89	5.09
LAMC2	8.58	1.35	3.32	6.42	2.57	3.00
ATP12A	3.03	1.52	4.81	6.41	5.35	5.96
PLAU	2.10	1.11	5.31	6.38	5.51	5.67
RGS4	2.06	1.00	5.20	6.25	0.00	0.00
ATP6V1C2	3.24	5.20	4.51	6.20	3.29	5.67
TTC18	2.00	1.03	5.18	6.18	6.10	6.14
PLA2G4C	3.15	1.84	4.51	6.16	4.20	5.09
ATP9A	5.36	2.29	3.74	6.16	0.00	1.19
TM4SF1	22.38	1.00	1.68	6.16	0.00	0.00
C2CD4A	2.70	1.30	4.64	6.07	1.94	2.32
DUSP6	2.02	1.35	4.88	5.89	5.56	5.99
ZBP1	2.18	1.43	4.77	5.89	5.41	5.92
CCDC168	2.19	1.00	4.73	5.86	6.44	6.45
MAP7	2.45	1.54	4.57	5.86	4.73	5.35
ULBP1	2.01	1.65	4.81	5.81	5.35	6.08
PIK3IP1	2.31	1.32	4.61	5.81	4.58	4.98
JPH3	2.47	1.18	4.51	5.81	5.77	6.01
PAPPA2	2.47	-1.55	4.51	5.81	4.52	3.89
WDFY3	2.02	1.27	4.73	5.75	4.28	4.63
CADPS	7.57	1.67	2.78	5.70	1.94	2.68
FLRT3	2.21	1.98	4.46	5.60	4.68	5.67
DIP2C	2.54	2.09	4.24	5.58	4.64	5.70
SAMD4A	2.54	1.59	4.24	5.58	4.58	5.25

ELF3	3.33	-1.11	3.85	5.58	3.74	3.60
FILIP1L	2.07	2.43	4.51	5.56	4.73	6.01
PRSS1	4.81	1.07	3.22	5.49	1.10	1.19
REG1B	2.32	1.00	4.24	5.45	0.00	0.00
ANK1	3.45	1.39	3.62	5.41	3.54	4.01
VGF	2.26	3.00	4.20	5.38	1.10	2.68
ELAVL4	2.35	1.48	4.12	5.35	4.52	5.09
GCNT3	3.30	-1.34	3.62	5.35	4.02	3.60
DNAJC12	2.07	1.42	4.24	5.29	4.36	4.86
CPB1	7.07	1.00	2.43	5.25	0.00	0.00
GAD2	3.23	2.29	3.54	5.23	0.00	1.19
PNLIP	6.98	1.00	2.43	5.23	0.00	0.00
RFX6	26.53	1.00	0.50	5.23	0.00	0.00
SSTR2	11.51	-1.23	1.68	5.20	2.98	2.68
HSPA6	4.43	-2.97	2.98	5.13	3.89	2.32
TJP1	2.75	3.33	3.62	5.09	1.48	3.22
TMEM200A	3.32	-2.14	3.32	5.05	1.10	0.00
DSG2	2.48	1.00	1.00	3.74	5.05	0.00
RPS15	-2.70	-1.46	8.55	7.11	6.50	5.96
CHCHD10	-2.11	1.54	6.43	5.35	6.23	6.85
SPP1	-2.25	1.22	6.30	5.13	6.42	6.71
TWF2	-2.05	-2.04	6.01	4.98	5.45	4.42
C16orf42	-2.13	1.62	5.97	4.88	4.82	5.51
CD320	-2.48	-1.16	5.97	4.66	4.64	4.42
FKBP8	-2.63	1.03	5.89	4.50	4.58	4.63
HIST1H4I	-2.61	-1.98	5.27	3.89	4.42	3.43
KIAA0415	-2.08	-1.39	5.18	4.12	4.36	3.89
MBD3	-2.61	-2.31	5.12	3.74	3.89	2.68
SSSCA1	-2.02	-1.29	5.04	4.02	4.79	4.42
INS	-8.34	1.00	5.00	1.94	0.00	0.00
