# Supplementary figures and images for: Dynamic Changes in Gene Mutational Landscape With Preservation of Core Mutations in Mantle Cell Lymphoma Cells
Source: Front Oncol. 2019 Jul 3;9:568. doi: 10.3389/fonc.2019.00568 (PMC6617136; doi:10.3389/fonc.2019.00568)

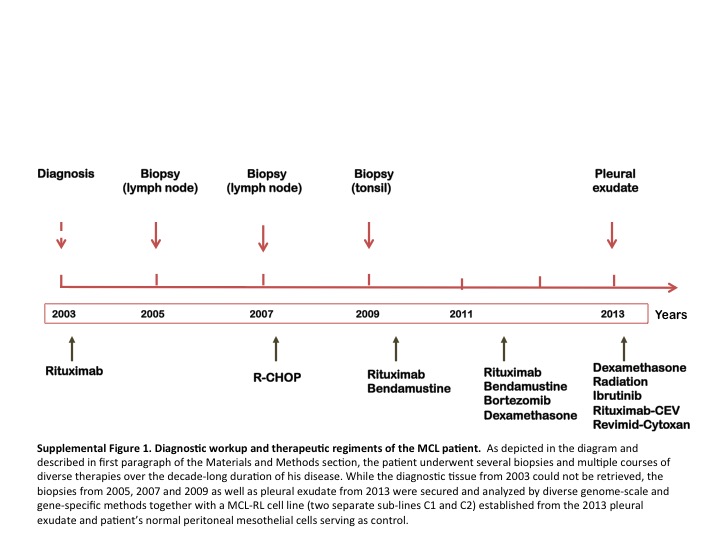

Supplement: Supplementary file 7 [file Image_1.jpeg]
