# Supplementary material for: Dynamic Changes in Gene Mutational Landscape With Preservation of Core Mutations in Mantle Cell Lymphoma Cells
Source: Front Oncol. 2019 Jul 3;9:568. doi: 10.3389/fonc.2019.00568 (PMC6617136; doi:10.3389/fonc.2019.00568)
Supplement: Supplementary file 8 [file Image_2.pdf]

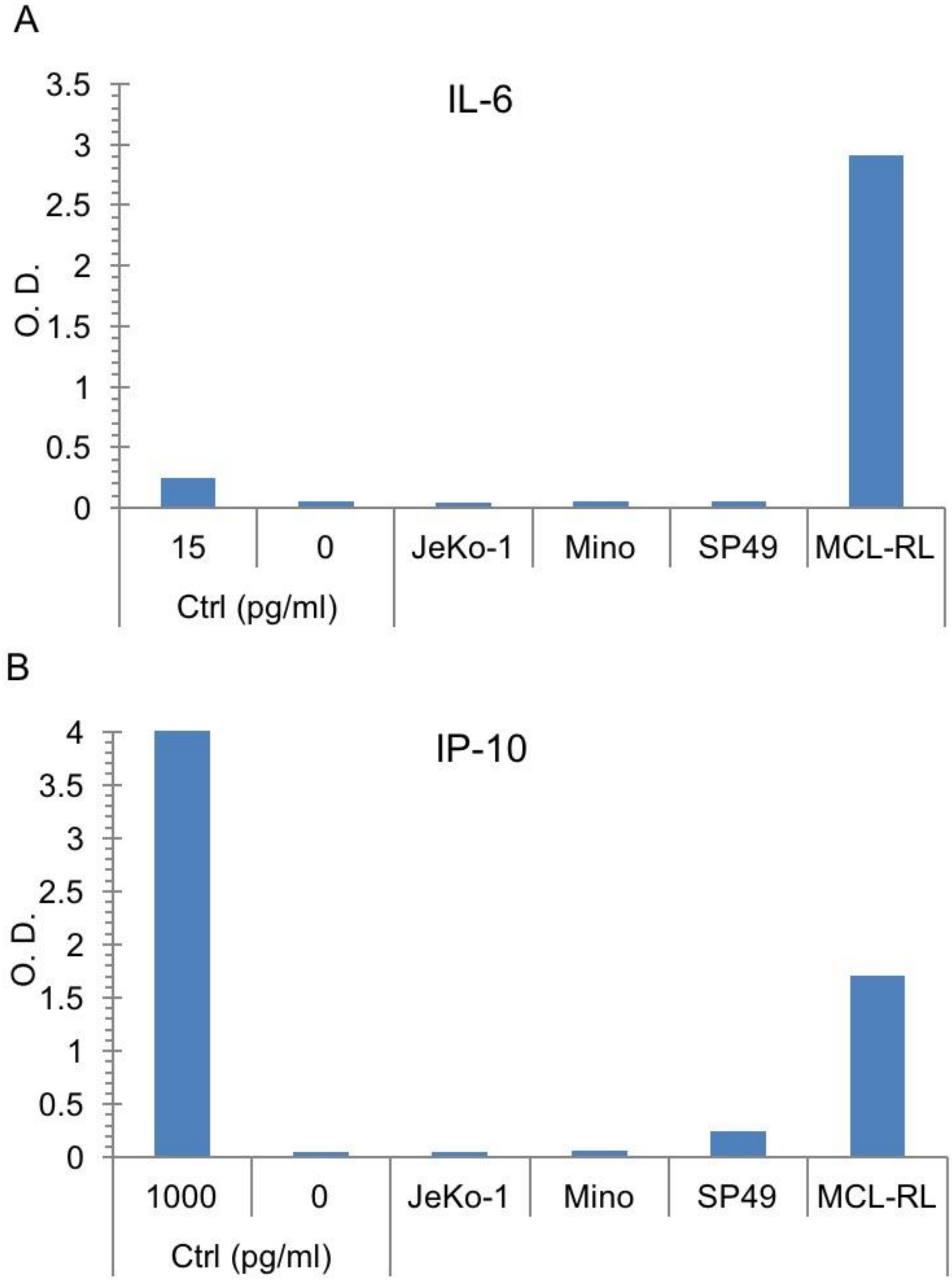

Supplemental Figure 2. Secretion of IL-6 and IP-10 by MCL-RL cells. Cytokine-specific enzyme-immunoassay (EIA) was used to detect expression of IL-6 (panel A) and IP-10 (panel B) in quadruplicate 24h culture supernatants of the depicted MCL cell lines. Standards provided by manufacturer's provided served as a positive and negative controls (Ctrl). The results are expressed as optical density (O.D.) values. Standard deviations for each quadruplicate set were <10%.
