# Supplementary material for: Dynamic Changes in Gene Mutational Landscape With Preservation of Core Mutations in Mantle Cell Lymphoma Cells
Source: Front Oncol. 2019 Jul 3;9:568. doi: 10.3389/fonc.2019.00568 (PMC6617136; doi:10.3389/fonc.2019.00568)
Supplement: Supplementary file 9 [file Image_3.pdf]

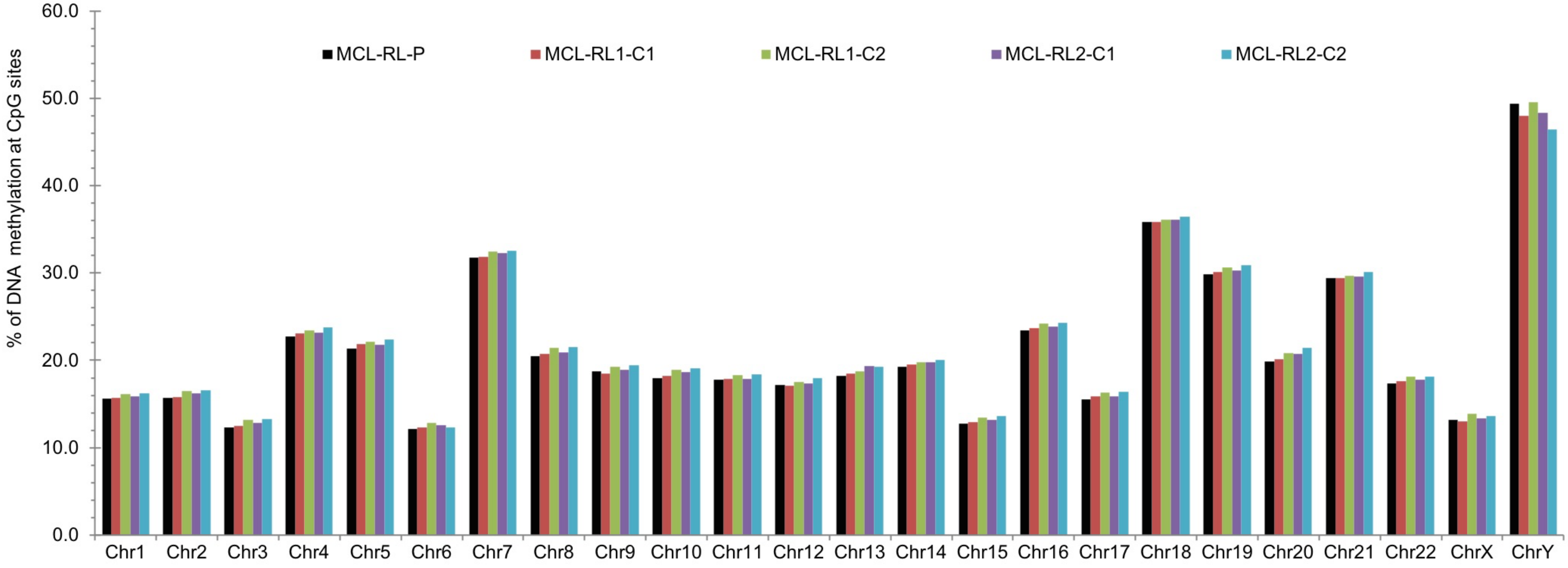

Supplemental Figure 3. DNA methylation pattern in primary and cultured MCL cells. Comparative analysis of whole genome DNA methylation in primary cells and primary cell-derived cultured MCL-RL cells. The results show methylation degree of all 23 chromosome pairs in the primary cells (MCL-RL--P), first sub-cell line maintained for 3 months (MCL-RL1-C1) and 6 months (MCL-RL1-C2) since initiation of fresh primary cells culture, and second sub-cell line also maintained for 3 months (MCL-RL2-C1) and 6 months (MCL-RL2-C2).
